# An optimized 3D-printed perfusion bioreactor for homogeneous cell seeding in bone substitute scaffolds for future chairside applications

**DOI:** 10.1038/s41598-021-01516-8

**Published:** 2021-11-15

**Authors:** Nadja Engel, Carsten Fechner, Annika Voges, Robert Ott, Jan Stenzel, Stefan Siewert, Carina Bergner, Valeria Khaimov, Jan Liese, Klaus-Peter Schmitz, Bernd Joachim Krause, Bernhard Frerich

**Affiliations:** 1 Experimental Research Laboratory, Department of Oral and Maxillofacial Surgery, Facial Plastic Surgery, Rostock University Medical Center, Schillingallee 35, 18057 Rostock, Germany; 2 Institute for Implant Technology and Biomaterials e.V, Friedrich-Barnewitz-Str. 4, 18119 Rostock, Germany; 3 Core Facility Multimodal Small Animal Imaging, Rostock University Medical Center, Schillingallee 69a, 18057 Rostock, Germany; 4 Radiopharmacy, Department of Nuclear Medicine, Rostock University Medical Center, Gertrudenplatz 1, 18057 Rostock, Germany; 5 Department of Nuclear Medicine, Rostock University Medical Center, Gertrudenplatz 1, 18057 Rostock, Germany; 6 Department of Oral and Maxillofacial Surgery, Facial Plastic Surgery, Rostock University Medical Center, Schillingallee 35, 18057 Rostock, Germany

**Keywords:** Biotechnology, Stem cells, Medical research, Materials science

## Abstract

A clinical implementation of cell-based bone regeneration in combination with scaffold materials requires the development of efficient, controlled and reproducible seeding procedures and a tailor-made bioreactor design. A perfusion system for efficient, homogeneous, and rapid seeding with human adipogenic stem cells in bone substitute scaffolds was designed. Variants concerning medium inlet and outlet port geometry, i.e. cylindrical or conical diffuser, cell concentration, perfusion mode and perfusion rates were simulated in silico. Cell distribution during perfusion was monitored by dynamic [^18^F]FDG micro-PET/CT and validated by laser scanning microscopy with three-dimensional image reconstruction. By iterative feedback of the in silico and in vitro experiments, the homogeneity of cell distribution throughout the scaffold was optimized with adjustment of flow rates, cell density and perfusion properties. Finally, a bioreactor with a conical diffusor geometry was developed, that allows a homogeneous cell seeding (hoover coefficient: 0.24) in less than 60 min with an oscillating perfusion mode. During this short period of time, the cells initially adhere within the entire scaffold and stay viable. After two weeks, the formation of several cell layers was observed, which was associated with an osteogenic differentiation process. This newly designed bioreactor may be considered as a prototype for chairside application.

## Introduction

There undoubtedly is a substantial need for new regenerative strategies in the management of craniofacial bone deficits, for example before implantation in dental surgery^1–3^. For these purposes, ceramic bone substitute materials have been clinically established and are widely used but allow only limited augmentation^4^. A successful bone formation takes place over a maximum gap distance of 2–3 mm within the bone, vertical and voluminous augmentations are associated with higher complication rates since bone has a limited capacity for spontaneous healing of critical defects caused by injury, inflammation or therapeutic resection^5,6^. It has been proven meanwhile in animal and clinical studies that the combination of osteogenic progenitor cells and bone graft substitutes can significantly increase their efficiency and regenerative value^7,8^. In the field of maxillofacial bone grafting, the group of Smiler et al. presented a study in which bone marrow aspirate containing adult stem cells was combined with bioengineered graft materials^9^. This combination improved the proliferation, differentiation, and maturation of the stem cells, as well as facilitating angiogenesis. Further, Soltan et al. conjure up that the addition of bone marrow aspirate, containing adult stem cells, to an osteoconductive matrix can contribute substantially to the efficacy of bone augmentation in the maxilla and mandible^10^.

There are, however, several barriers to those approaches, including their costs, the complexity and the regulatory issues^11^. These limitations have led to different concepts including the use of chairside methods^12^. Stem cells that are isolated from the adipose tissue are best suited for this chairside applications, since they are easily to harvest and in most cases the source is inexhaustible^13–15^.

Regardless of which approach is used, devices and methods are required to ensure effective, homogeneous, and reproducible seeding of scaffolds or carrier materials. A clinically acceptable seeding system should be self-contained, and the process should be completed within a defined short period of time^16–18^. Moreover, a homogeneous seeding should also be feasible with different scaffold geometries, e.g., if thinking of individualized alveolar ridge augmentation.

In terms of quality control, the homogeneity of cell seeding must be easily verifiable^19^. This represents a special challenge for opaque ceramic scaffolds—optical methods are inapplicable, histological methods for serial examinations are time-consuming and laborious. Using the combination of positron emission tomography (PET) and computed tomography (CT) within a micro-PET/CT, [^18^F]FDG-labeled stem cells can be monitored during perfusion processes to assess both, the colonization in three dimensions and the vitality of the cells within opaque scaffolds. Here, we present a method in which a bioreactor specifically designed for seeding of ceramic bone blocks can deliver reproducible and efficient results for given scaffold geometries. Approved scaffold materials in the form of deproteinized cancellous bovine bone grafts and human adipogenic stem cells were used as a model situation. In order to save experiments, virtual computational modeling was used beforehand, and the results of the modeling were iteratively compared with the experiment.

## Material and methods

### Mathematical modeling of cell seeding in xenograft blocks

A clinically established and FDA approved bone substitute material was used as a model for all the simulations and experiments (Bio-Oss® Block, Geistlich Pharma AG, Switzerland). According to the manufacturer, it is composed of deproteinized and sterilized cancellous bovine bone with a cuboid dimension of 10 mm × 10 mm × 20 mm, a bimodal pore structure with mean pore diameters of d1 = 17 nm and d2 = 1.07 × 10^5^ nm and a porosity of 69%^20^. Xenograft blocks are physically and chemically comparable to the mineralized matrix of human bone rendering by osteoconductive properties and suitable for bone regeneration.

**Figure 1:**
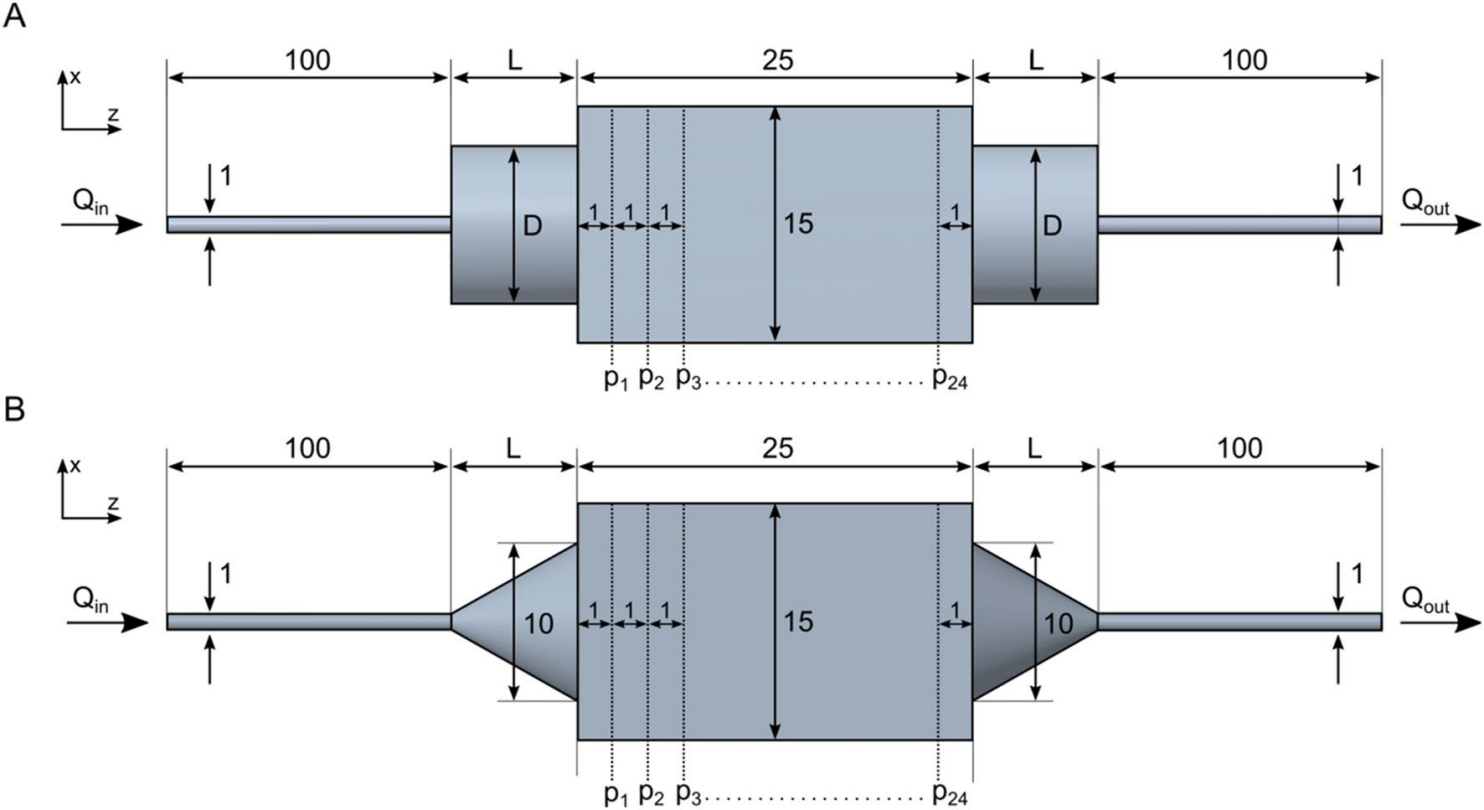
Schematic representation of numerically investigated cylindrical (**A**) and conical (**B**) connector geometries. For the cylindrical geometry, the influence of different connector diameters (D1 = 1 mm, D2 = 10 mm) as well as lengths (L1 = 1 mm, L2 = 2 mm, L3 = 3 mm, L4 = 4 mm, L5 = 5 mm, L6 = 10 mm) was investigated. For the conical connector geometry, the influence of different connector lengths (L1, L2, L3, L4, L5, L6) with a constant connector diameter (D2) was investigated. The Hoover coefficient was calculated at 24 cross-sectional planes perpendicularly aligned to the longitudinal axis of the scaffold.

The 3D scaffold geometry of Bio-Oss blocks was obtained by means of micro-computed tomography (Skyscan 1172, Bruker microCT, Belgium) at a resolution of 9.5 μm. Computational fluid dynamics (CFD) analysis (ANSYS Fluent, ANSYS Inc., Canonsburg, PA, USA) of fluid flow in xenograft blocks were conducted as already described^20^. Briefly, for the numerical simulations steady-state flow conditions, an inlet flow rate of 1 ml/min, an outlet pressure of 0 Pa and no-slip-conditions on the walls were applied. Based on experimental investigations at 37 °C using a Haake RheoStress 1 rotational rheometer equipped with a double cone setup (Thermo Fisher Scientific Inc., Newington, NH, USA), dynamic viscosity and density of the cell suspension (ρ = 1 × 10^6^ cells/ml) were set to 1.176 mPas, and 1004 kg/m^3^ respectively. In the numerical simulations, the necessity of a chamber with a form-locking fit with respect to the scaffold block was postulated. Connector geometries for the inflow and outflow of the perfusate were simulated and compared with regard to the homogeneity of flow within the scaffold. Firstly, the influence of different diameters (D_1_ = 1 mm, D_2_ = 10 mm) of the cylindrical connector geometry was investigated (Fig. 1A). Secondly, cylindrical and conical connector geometries were simulated with in- and outflow lengths ranging from L = 1 mm to L = 10 mm, respectively (Fig. 1A, B). To quantitatively compare the homogeneity of the flow through the different bioreactor designs, the Hoover coefficient was calculated at 24 cross-sectional planes with a constant spacing of 1 mm (Fig. 1A, B).

### Design and manufacture of bioreactors

**Figure 2:**
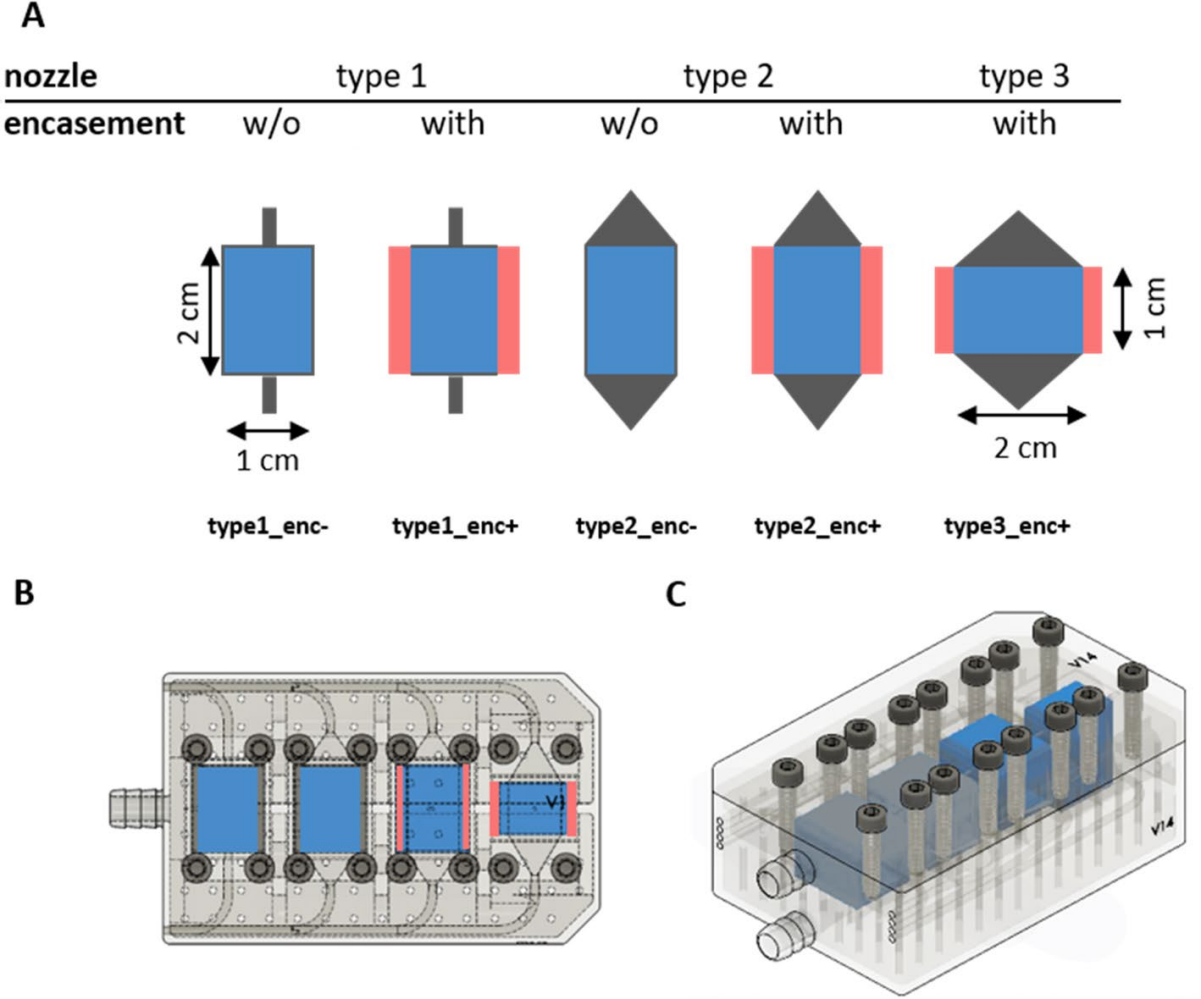
Bioreactor prototypes. (**A**) Schematic presentation of the different bioreactor types (longitudinal section). Black: shape of the perfusion chamber and the inflow/outflow nozzles. Blue: scaffold block. Red: silicone encasement around the scaffold. The perfusion chambers of type 1 and type 2 are 1 cm in width and 2 cm in height, type 3 is 2 cm in width and 1 cm in height. The presence or absence of the encasement is indicated by enc+ or enc−, respectively. (**B**) Design of the four bioreactors integrated in a single 3D-printed block drawn by Autocad Fusion 360. The different inlet and outlet geometries are visible above and below the scaffold chambers. Arrows indicate separate inflow and outflow lines of the chambers. Central connectors are needed for the water circulation system to regulate the temperature. Bioreactor types 2 and 3 with encasement harbor a silicone sealing (red) for the scaffolds (blue). (**C**) 3D view of the bioreactor without the silicon encasement.

The design of the perfusion chambers was developed using Autocad Fusion 360 (Autodesk, Inc., CA, USA). Perfusion chambers were printed with a Formlabs Form2 3D printer (Formlabs Inc., MA, USA) using the Dental SG 1 L resin (class 1 biocompatible synthetic resin (EN-ISO 10,993–1:2009/AC:2010, USP class 4) by stereolithography. A layer thickness of 25 µm was used. The dimensions of the perfusion chambers are 1 cm in width, 2 cm in height and 1 cm in depth, which guaranteed that the Bio-Oss blocks perfectly matched to the chambers. Type 3 of the chambers has the same dimensions, was only integrated horizontally into the perfusion module. The chamber closing mechanism was ensured by a conventional rubber seal on the module cover, which was firmly closed with the perfusion module using 4 screws per perfusion chamber (Fig. 2C). The biocompatible silicone tubing material for flow applications was purchased by ibidi GmbH, Gräfelfing, Germany (#10841). All perfusion related materials were preheated at 37 °C in 5% CO_2_ and 95% air-humidified incubator at least for 24 h.

Based on the results of the numerical simulations, different connector geometries were applied using different types of lateral encasement and orientation of the scaffold (Fig. 2A). The following connector dimensions for inflow and outflow were used: (i) 1 mm cylindrical shaped connector geometry (type 1) as well as (ii) conical shaped connector geometry with 15 mm in height x 15 mm in width (type 2) and 15 mm in height × 25 mm in width (type 3). The conically shaped connectors (type 2, 3) are firmly attached to the scaffold housing, so that the cell inflow is made possible over the entire upper scaffold surface. On the other hand, the cylindrical connector only allows punctual inflow through the 1 mm wide opening.

In the experimental investigations of type 1 and 2 connector geometries, the scaffold was perfused lengthwise (along the longitudinal axis). In contrast, the scaffold of type 3 connector geometry was transversally perfused to its longitudinal axis. Whereas the perfusion chamber itself was initially designed loose-fit to the scaffold block (type1/2_enc−), it was equipped with a press-fit encasement to the scaffold block (type1/2/3_enc+) in later experiments manufactured from dental grade silicone (Wagnersil 9N, Wagner Dental, Hückelhoven, Germany) with a Shore Hardness of 9 to 35 Shore. It was individually prepared to fit inside the perfusion chambers and tightly enclose the scaffold block, allowing the direct perfusion through the scaffold and preventing a flow over the lateral surfaces. For this purpose, a template of the scaffold was created by 3D printing to cast the silicone encasement around the scaffold. After curing and sterilization, the scaffold was placed directly into the silicone encasement.

For the micro PET/CT experiments, four perfusion chambers were integrated in a single 3D-printed bioreactor block in order to perform multiple experiments and measurements simultaneously in a single PET/CT session (Fig. 2B). Perfusion was realized by negative pressure from the outflow side by NE-4000 double syringe pump (modified for up to four syringes; New Era Pump Systems Inc., East Farmingdale, NY, USA) or Ibidi Pump System (ibidi GmbH, Gräfelfing, Germany). The cell suspension was gently stirred in a 250 ml flask and was routed via individual feed lines to the inflow side of each perfusion chamber. Each perfusion chamber in the bioreactor block was separately controlled by one pump/syringe. To maintain constant temperatures in the micro PET/CT experiments, a temperature-control system based on a water circulation system operated by Medres heated pump and equipped with a Carel Ir33 temperature controller (Carel Industries S.p.A., Brugine, Padova, Italy) was implemented. As oxygen sensor, the PreSens O2 Flow-Through Cell FTC-PSt3 in conjunction with the PreSens Oxy 10 (PreSens Precision Sensing GmbH, Regensburg, Germany) was used to monitor oxygen content within the bioreactor. One sensor each was placed at the inlet and outlet of the connectors. A representation of the bioreactor setup in combination with the perfusion pump is shown in Supplementary Fig. 1.

### Isolation, propagation and characterization of adipose-derived stem cells (ASC)

Lipoaspirate samples were collected from patients undergoing liposuction or lipofilling procedures at the Rostock University Hospital or Plastic Surgery Clinic in Rostock, Germany, with approval from the Ethics Committee at the University Medical Center Rostock No. A 2014–0092. All patients provided informed consent. The procedure for isolating adipose-derived stem cells (ASC) has been described elsewhere^21^. Briefly, the samples were washed with PBS, and transferred in a 50 ml Falcon Tube containing 10 ml PBS with 6 mg/ml collagenase NB4 (SERVA Electrophoresis GmbH, Heidelberg, Germany). After 30 min tissue digestion using a Lab Rotator at 37 °C, the digestate was filtered through a 100 μm cell strainer (Becton Dickinson, Franklin Lakes, NJ, USA) by adding 10 ml PBS including 10% Hyclone Newborn Calf Serum (NCS; Sigma Aldrich Chemie GmbH, Munich, Germany) and was finally centrifuged at 1000 rpm for 10 min. The pellet was washed in 10 ml PBS/10% NCS, centrifuged again and resuspended in 10 ml PBS/10% NCS. Adipose-derived stromal cells were kept at 37 °C in 5% CO_2_ and 95% air-humidified incubator. Cell culture medium constituted of 45% Iscove’s Modified Dulbecco's Medium, 45% Gibco® F-12 Nutrient Mixture, 10% NCS and supplemented with 0.1 ml Gibco™ Penicillin–Streptomycin (all from Thermo Fisher Scientific GmbH, Regensburg, Germany) and 10 µg basic fibroblast growth factor (recombinant human bFGF; Millipore Merck KGaA, Darmstadt, Germany) per 1 l of cell culture media. Once 80% confluency was reached, flasks were passaged in a 1:3 ratio. Adherent ASCs were assessed for mycoplasma contamination using a PCR-based assay and DAPI staining (Life Technologies GmbH, Darmstadt, Germany). The ASCs were used up to the 3rd passage. The ability for adipogenic, osteogenic, and chondrogenic differentiation of these ASCs as evidence of mesenchymal stem cell character has been described elsewhere^22,23^. Moreover, expression of CD73, CD90, and CD105 was proven by flow cytometry using BD FACSAria™ IIIu (BD Biosciences, San Jose, CA, USA). Therefore, all primary antibodies (HLA-DR, CD105, CD90, CD45, CD73, CD34, CD19, CD14) were purchased from BD Biosciences and results were calculated using FlowJo (https://www.flowjo.com).

### Comparison of cell concentrations and perfusion rates

Prior to micro-PET/CT experiments, a first series of experiments with cell imaging by confocal laser-scanning microscopy (LSM) was performed in order to test optimal seeding parameters like cell concentration and perfusion rate using the bioreactor type2_enc+ . Prior to perfusion, stem cells were labeled with the PKH26 Red Fluorescent Cell Linker Kit (Sigma-Aldrich Chemie GmbH) following manufacturer’s instructions. Either the unidirectional or the oscillating perfusion mode was selected. Unidirectional means that the cells were only perfused through the scaffold from top to bottom. In the oscillating mode, the orientation was switched every 5th minute. The perfusion chambers including scaffold blocks were perfused with warmed cell suspension (37 °C) with a priming volume of 10 ml over 60 min and washed with warmed PBS (w/o Ca^2+^ and Mg^2+^; Biochrom GmbH, Berlin, Germany) thereafter. First, perfusion was performed with cell concentrations between 0.06 and 1.22 × 10^6^ cells/ml at a constant perfusion rate of 0.5 ml/min in order to check the influence of the cell concentration on the seeding efficiency and homogeneity (10 steps). In a second series, the influence of the perfusion rate was tested by using 0.25, 0.5, 1.0, and 2.0 ml/min at a constant cell concentration of 0.4 × 10^6^ cells/ml (n = 3). After retrieving the scaffolds, bioreactors and tubes were washed with 100% alcohol (cleaning solution). Number of residual cells was measured in the outflow, the wash out, and the cleaning solution. Results were related to the inflow concentration to calculate the seeding efficiency.

### LSM imaging and 3D image reconstruction

Confocal laser-scanning microscopy (LSM) was used to analyze the three-dimensional (3D) spatial distribution of cells within the scaffold blocks to check calculated seeding efficiency. After cell perfusion, scaffolds were fixed in 4% paraformaldehyde (Santa Cruz, Dallas, USA), washed with PBS, and cut in into 1.5 mm slices using an Exakt Diamant Bandsäge 300 CL saw (EXAKT Advanced Technologies GmbH, Norderstedt, Germany). PKH26-labeled cells were investigated with an inverted confocal laser-scanning microscope (LSM780, Carl Zeiss Microscopy GmbH, Jena, Germany). The following fixed settings were used for scanning: 40 µm, 3.2 AU, Alexa Fluor555, resolution 1024 × 1024 Pixel per tile, 10 × magnification Tiles (20 × 20). Overlay images of z-scans were assembled by scanning the cells on the surface at 0.8 µm intervals (software ZEN black, Carl Zeiss Microscopy GmbH). 3D reconstruction of the scaffold blocks was generated with the software Fiji ImageJ (https://imagej.net/Fiji/), a self-written program and visualized with VisIt (https://visit.llnl.gov/) (Supplemental Fig. 2). The distribution of the fluorescence within the 3D reconstructed blocks served as a qualitative measure for the homogeneity of the seeding.

### [^18^F]FDG micro-PET/CT imaging

[^18^F]FDG-labeling and micro-PET/CT examination was used in the main series of experiments for the real-time imaging of cell distribution in the scaffold blocks. Prior to this, [^18^F]FDG uptake of ASC was tested up to 180 min after administration of [^18^F]FDG. 2 × 10^5^ ASCs of the 3^rd^ passage were trypsinized, centrifuged, and resuspended in 1 ml cell culture media. [^18^F]FDG has been synthesized fully automated at the GMP laboratory of the Department of Nuclear Medicine using a Fastlab module in combination with corresponding FDG-citrate cassettes (GE Healthcare). The required [^18^F]fluorine has been obtained from the in-house cyclotron MiniTrace PT 700 (GE Healthcare). Cells were incubated with 1 MBq [^18^F]FDG at 37 ℃ under continuous stirring. After 10, 30, 60, 90, 120 and 180 min, cells were washed and trypsinized followed by repeated washing steps with PBS. Residual activity of the cells was measured using a gamma counter (WIZARD2 10 detector Gamma Counter; Perkin Elmer, Waltham, MA, USA). The measurements were repeatedly performed (n = 6). Based on these results (Supplementary Fig. 3), an incubation time of 60 min was considered to be adequate for [^18^F]FDG-labeling, because no significant differences in [^18^F]FDG-uptake were calculated after 60 min, and used in the following perfusion experiments. [^18^F]FDG-labeled cells were washed twice with PBS and diluted to a concentration of 0.4 × 10^6^ cells/ml. Perfusion of scaffold blocks in the bioreactors was conducted as described above and imaging studies were performed using a preclinical micro-PET/CT system (Inveon®, Siemens Healthcare Erlangen, Germany). A different number of experiments were performed depending on the bioreactor type: loose-fit perfusion chambers without silicone encasement (bioreactor type 1 (n = 1) and bioreactor type 2 (n = 2)), bioreactors with silicon encasement (bioreactor type 1 to 3 (n = 3 each) and bioreactor type 3 with oscillating perfusion direction (n = 3). The total PET scan time was approx. 100 min including unidirectional perfusion with [^18^F]FDG-labeled stem cells in the first 60 min. For further 40 min, seeded scaffolds were observed during a wash-out-phase with PBS to clean the bioreactor of remaining free FDG and non-adherent cells. In case of bioreactor type 2 with encasement, an additional oscillating mode was tested with changing the perfusion direction once after 30 min (type2_enc+ _osc).

Each PET data set was corrected for random coincidences, dead time and attenuation. PET images with dynamic framing every 5 min were reconstructed using a 3D Ordered Subset Expectation Maximization with Maximum A Priori Shifted Poisson algorithm (3DOSEM / SP MAP). For quantitative results, regions of interest (ROI) were placed around the whole scaffold block, in the upper, middle and lower third as well as in the outflow tract to keep track of cell sedimentation outside the scaffold. Data were averaged and cell distribution was compared regarding bioreactor types and perfusion algorithms. The Hoover coefficients (0 representing total equal distribution and 1 representing maximal inequality) were calculated for every bioreactor type as a measure of the inhomogeneity of cell distribution within the scaffold block. In order to validate calculated Hoover indices using [^18^F]FDG-labeled cells, results were compared to 3D reconstructions from LSM evaluation using PKH26-labeled cells. Both experiments were performed with a bioreactor type2_enc+ . In addition, cell distributions within the scaffold blocks were compared to the in silico calculations generated from numerical simulations.

### Cell adherence in scanning electron microscopy (SEM)

To investigate cell adherence after perfusion, scaffold blocks were incubated in culture medium for 3, 6 or 24 h after the one-hour perfusion (t_0_) with 0.4 × 10^6^ cells/ml in the bioreactor type 2 with encasement. Scaffold blocks were washed with PBS, fixed with 2.5% glutaraldehyde in 0.05 M HEPES buffer, dehydrated in an ascending ethanol series and critical point dried using CO_2_ as an intermedium with the EMITECH 850 critical point dryer (Emitech Ltd., Ashford, UK). Carbon coating was done with the carbon coater SCD500 (Leica Microsystems GmbH, Wetzlar, Germany). Images were taken with a QUANTA FEG 250 field emission scanning electron microscope (FEI Company, Hillsboro, OR, USA) using an acceleration voltage of 5–10 kV and a sample chamber pressure of *p* < 1.5 × 10^–2^ Pa (‘high vacuum’ mode) for imaging.

### Long-term perfusion and initial osteogenic differentiation

For the long-term experiments, Bio-Oss blocks were perfused in bioreactor type 2_enc+ with 0.4 × 10^6^ cells/ml for 60 min using the Ibidi pump system (ibidi GmbH). An oscillating perfusion rate of 0.5 ml/min was chosen. After perfusion, scaffolds were incubated in a roller culture flask for 14 days and the cell culture medium was changed every three days. All experiments were performed in triplicates. The initial osteogenic differentiation was analyzed by Alizarin red staining according to manufacturer's protocol (Osteogenesis assay kit, #ECM815, Millipore, Burlington, MA, USA). Briefly, cells were washed with PBS, fixed with 4% paraformaldehyde for 10 min, washed again, and incubated with 0.1% Alizarin red for 30 min. Finally, cells were washed until red stained structures were clearly visible. Visualization and imaging were carried out with the Axioscope A1 microscope using AxioVision Imaging Software 4.8.2.0 (both from Carl Zeiss Microscopy GmbH).

### Immunohistochemistry (IHC)

After cell perfusion, scaffold blocks were fixed in 4% buffered formalin (Formafix®, Global Technologies Ltd., Düsseldorf, Germany) further dehydrated and infiltrated with embedding resin (Technovit 9100, Kulzer, Wehrheim, Germany), cut to 200 µm slices, grind to 35–50 μm with sandpaper discs (1200 grit, EXAKT micro-grinding system), and final-polished with 4000 grit polish paper (EXAKT Advanced Technologies GmbH, Hamburg, Germany). These slices were stained with primary antibodies against collagen I (#ab34710), osteopontin (#ab8448), and bone sialoprotein (#ab52128; all from Abcam, Cambridge, UK). Secondary labeling was performed using an Alexa Fluor 488 goat anti-rabbit IgG (Thermo Fisher Scientific) combined with Hoechst counterstaining (PanReacAppliChem, Darmstadt, Germany). Stainings were investigated using the LSM780. Notably, images were taken at identical device settings to guarantee comparable results. The image processing was carried out using ZEN 2011 and 3D picture reconstruction was realized by taken z-scans with increments of 0.5 µm using ZEN black (both from Carl Zeiss Microscopy GmbH). A part of the sections of each block were treated with 10% hydrogen peroxide for 10 min after embedding in Technovit 7200 instead of 9100. Sections were stained with Giemsa’s azur eosin methylene blue solution (#109204; Merck KGaA, Darmstadt, Germany) for 30 min and toluidine blue-methylene blue-pyronin solution (#12796; Morphisto, Frankfurt (Main), Germany) for 10 min. Slides were scanned with an Axio Imager.M2 microscope equipped with an AxioCam MRc5 camera (both from Carl Zeiss Microscopy GmbH) that was connected to an automatic scanning table (M-686K011, Wienecke & Sinske GmbH, Gleichen, Germany).

### Statistical analysis

Statistical analyses were performed with GraphPad Prism 5 software (GraphPad Software, San Diego, CA, USA). Regarding cell enrichment and cell distribution, differences in [^18^F]FDG activity and Hoover coefficient were assessed using student’s t-test, respectively, and considered statistically significant at *P*-values of *P < 0.05, **P < 0.01, ***P < 0.001. In case of [^18^F]FDG uptake experiments, normal data distribution was tested by Kolmogorov–Smirnov test followed by Kruskal–Wallis one-way analysis of variance and Mann–Whitney U-test as *post-hoc* test. According to not normally distributed data, graph displays box-and-whisker diagram. Boxes include 25th and 95th percentiles as well as the median. Whiskers represent 10th and 90th percentiles. Pearson correlation coefficient (R^2^) was calculated to test linear correlation between seeding efficiency and cell concentration/perfusion rate. A correlation coefficient of -1 demonstrates a total negative linear correlation, whereby 0 represents no linear correlation.

## Results

### Simulation and modeling of bioreactor geometries in silico

**Figure 3:**
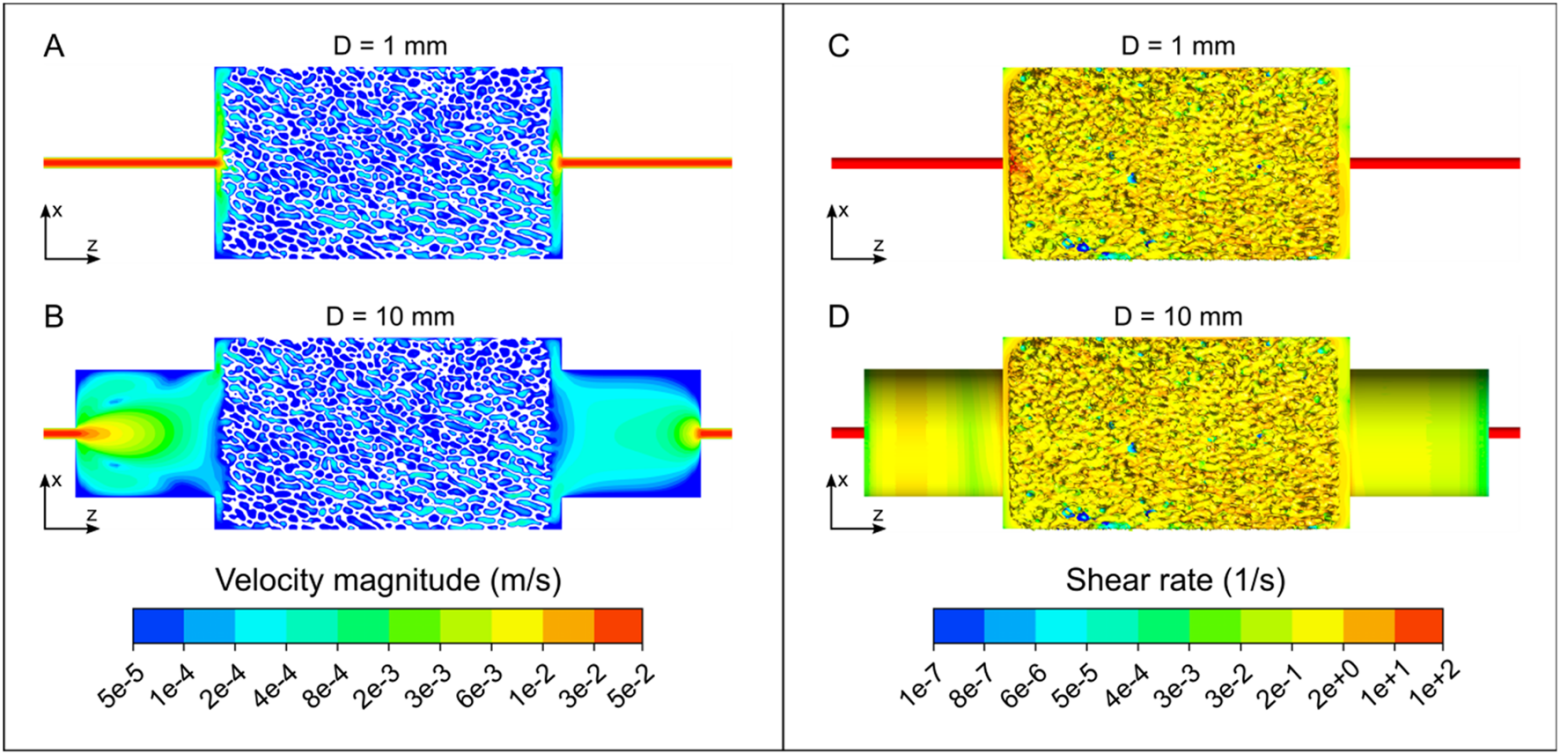
Numerically determined velocity magnitudes in a sectional plane (**A**, **B**) and shear rates in a cut view (**C**, **D**) of the bioreactor-scaffold-system with respect to the connector diameters (D = 1 mm, D = 10 mm).

In order to allow for a homogenous stem cell distribution, the geometry of the bioreactor was optimized so that a laminar, equally distributed volume flow throughout the bioreactor-scaffold system was achieved. In comparative in silico simulations, the homogeneity of the volume flow was quantified by the Hoover coefficient H_K_. The Hoover coefficient was calculated at 24 different sectional planes within the scaffold with a constant spacing of 1 mm perpendicularly aligned to the horizontal axis of the scaffold (Fig. 1). The numerical simulations revealed that the connector geometry at the inlet and outlet of the bioreactor had a decisive influence on the homogeneity of the flow within the scaffold. An increase of the diameter of the cylindrical connector from 1 to 10 mm improved the homogeneity of the flow in the scaffold by approx. 28% (reduction of the mean Hoover coefficient $\overline{{H_{K} }}$ from 0.6205 to 0.4441) (Fig. 3). In addition, the maximum shear rate inside the scaffold block was reduced from $\dot{\gamma }$_*max*_ = 49 s^−1^ to $\dot{\gamma }$_*max*_ = 18 s^−1^ and thus by approx. 63% by increasing the inlet and outlet diameters (Fig. 3C).

Further parameter studies showed that a shortening of the connector length from 10 to 1 mm led to a pronounced velocity maximum in the proximal and distal part of the scaffold resulting in a higher inhomogeneity for both connector geometries (increase from $\overline{{H_{K} }}$ = 0.4441 to $\overline{{H_{K} }}$ = 0.5973 for the cylindrical connector and from $\overline{{H_{K} }}$ = 0.5619 to $\overline{{H_{K} }}$ = 0.4301 for the conical connector) (Table 1).

**Table 1:** Mean Hoover coefficient of fluid flow in scaffolds with cylindrical and conical connector geometries with varying connector lengths.

Connector length	Cylindrical connector geometry	Conical connector geometry
L = 1 mm	0.6073	0.5571
L = 2 mm	0.5208	0.4915
L = 3 mm	0.4911	0.4542
L = 4 mm	0.4665	0.4075
L = 5 mm	0.4594	0.4238
L = 10 mm	0.4441	0.4301

**Figure 4:**
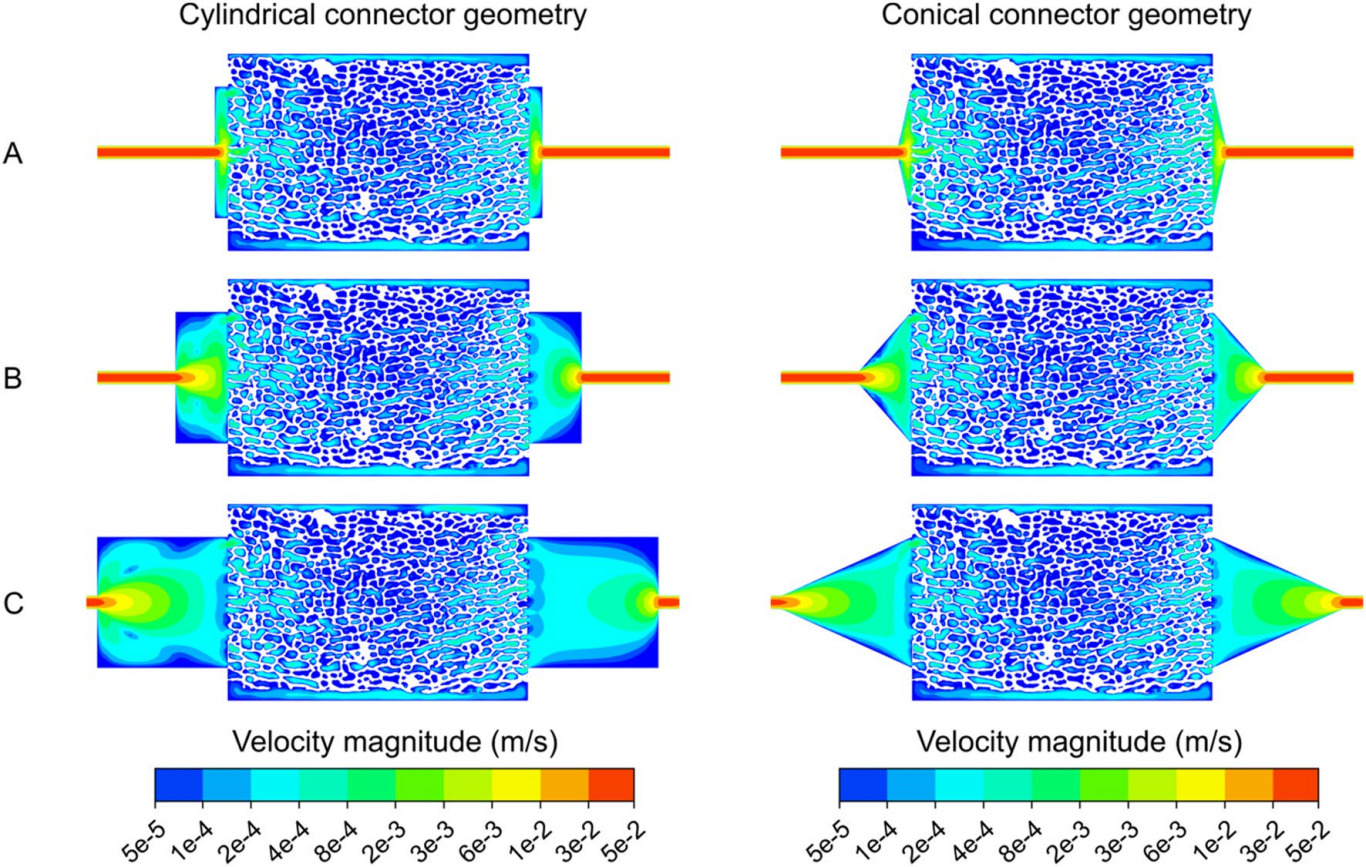
Numerically determined velocity magnitudes (logarithmic scale) in a sectional plane of the bioreactor-scaffold system with respect to the connector geometries and lengths (**A**) L = 1 mm, (**B**) L = 4 mm, and (**C**) L = 10 mm. An increase in connector length results in a clear reduction of velocity magnitude in central areas of the flow field.

This velocity maximum in the central area of the flow field decreased significantly with increasing length of the connector geometries at the inflow and the outflow (Fig. 4). A further improvement was achieved utilizing conical connector geometries on both sides. The conical connector arrangement resulted in consistently lower Hoover coefficients, leading to a more homogeneous flow through the bioreactor system compared to the cylinder inflow/outflow arrangement (Table 1, Fig. 4). However, while the Hoover coefficients for the cylindrical and conical connector inflow/outflow arrangement at a connector length of 1 mm differ by more than 8%, this difference decreases to 4% at a connector length of 10 mm. The most homogeneous flow of all investigated connector designs was achieved with the conical connector geometry with a length of 4 mm and a diameter of 10 mm ($\overline{{H_{K} }}$ = 0.4075).

### Testing of optimal perfusion conditions

**Figure 5:**
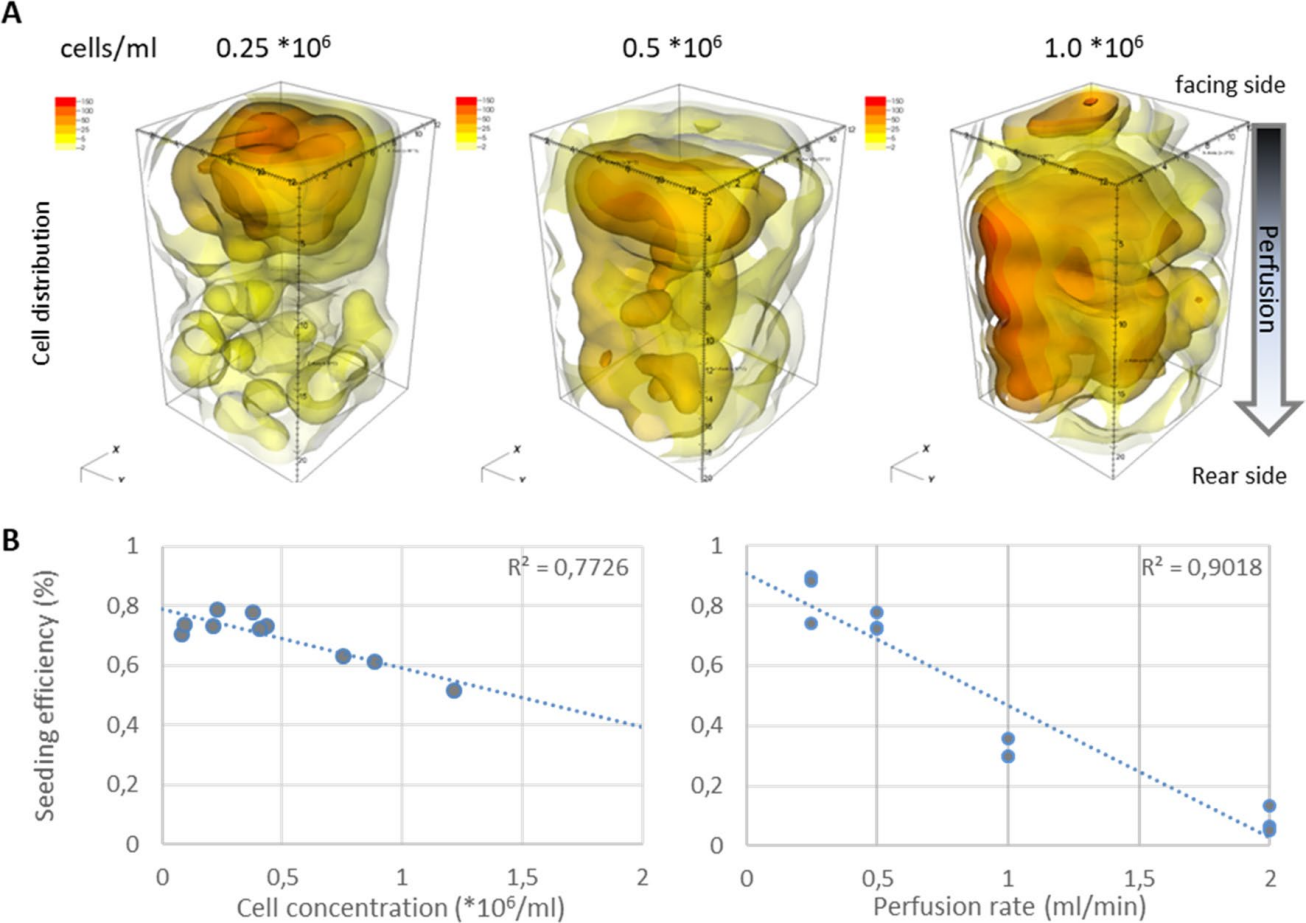
Influence of cell concentrations and perfusion rates on the cell distribution and homogeneity using bioreactor type2_enc+. (**A**) 3D reconstruction of perfused scaffold blocks using cell concentrations between 0.25 to 1.0 × 10^6^ cells/ml. Obviously, cells tend to accumulate in the upper half of the scaffold by using unidirectional perfusion. (**B**) Calculation of the cell seeding efficiency in dependence of the cell concentration (left, n = 3) and the perfusion rate (right, n = 3). Finally, low perfusion rates and moderate cell concentrations result in more efficient cell seeding of the scaffold.

For testing optimal seeding parameters like cell concentration and perfusion rate, the bioreactor type2_enc+ was used. Due to examples of fluorescence imaging on initially cut and then 3D reconstructed blocks, it is already evident that the higher the cell concentration in the perfusion solution, the higher the cell enrichment within the scaffold. (Fig. 5A). Using 0.25 × 10^6^ cells/ml, only the first third of the block was efficiently enriched with cells. Seeding of the lower half of the block was achieved through an increase in cell concentration to 0.5 × 10^6^ cells/ml. But only by using a cell concentration of 1.0 × 10^6^ cells/ml, the cylindrical bioreactor was seeded with ASC in the upper as well as in the lower area.

As shown in Fig. 5B, the seeding efficiency represents a percentage of cells deployed for perfusion. Higher cell concentrations in the perfusate lead to an increase in cell density within the scaffold. Considering the seeding efficiency as a function of the cell concentration in the suspension, a negative, almost linear correlation between the seeding efficiency and the cell concentration was observed at a given flow velocity of 0.5 ml/min and perfusion time of 60 min. At a concentration of 0.5 × 10^6^ cells/ml, approx. 70% of cells were found in the scaffold blocks, compared to only approx. 60% of cells by using 1 × 10^6^ cells/ml. An optimum of 80% seeding efficiency was achieved with a cell concentration of equal or less than 0.4 × 10^6^ cells/ml (R^2^ = − 0.7726, Fig. 5B left diagram). The perfusion rate, however, had a stronger influence on seeding efficiency: at a given cell concentrations of 0.4 × 10^6^ cells/ml suspension, the seeding efficiency fluctuated between 90% at below 0.5 ml/min and approx. 5% at 2 ml/min. According to these results, a strong negative linear correlation was proven (R^2^ = − 0.9018, Fig. 5B right diagram). For our purpose, an optimal seeding efficiency was achieved for 0.5 ml/min and 0.4 × 10^6^ cells/ml and used for further experiments.

### Validation of the [^18^F]FDG micro-PET/CT-based approach

**Figure 6:**
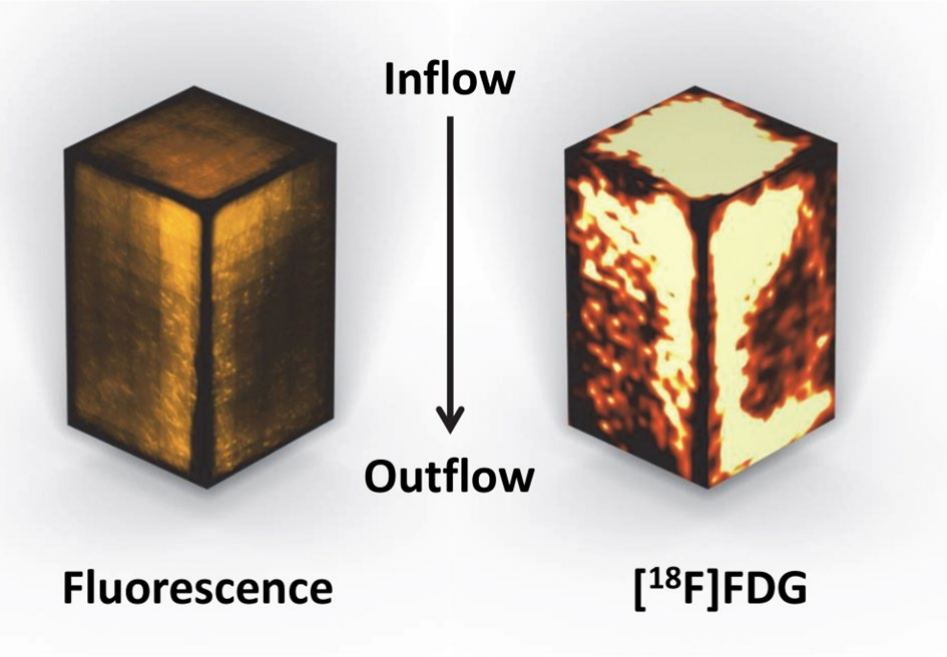
Comparison of fluorescence or [^18^F]FDG-labeling of ASC for imaging the cell distribution within the scaffold block. Both techniques show similar stem cell distribution patterns in the scaffold. Therefore, both are suitable for imaging of cell seeding.

Results of the first micro-PET/CT trials were compared to those of the LSM evaluation; in both approaches the bioreactor type2 with encasement (type2_enc+) had been used. The aim was to validate the newly established [^18^F]FDG micro-PET/CT approach by using the conventional fluorescence-based images from the experiments described above. The PET-based representation of the cell distribution in the scaffold block turned out to be congruent with that of LSM imaging (Fig. 6). PET-based imaging also showed an accumulation of activity (cells) in the proximal third of the block (at the inflow), a running along preferably one side of the block and a rather empty distal third of the block (at the outflow). Thus, micro-PET-based imaging proved to be suitable to replace the time-consuming LSM. In addition, it was shown that the mathematical modeling was able to give a realistic assessment of the flow conditions in the block and thus the cell distribution prior to the actual experiments in vitro.

### Comparison of different bioreactor configurations

**Figure 7:**
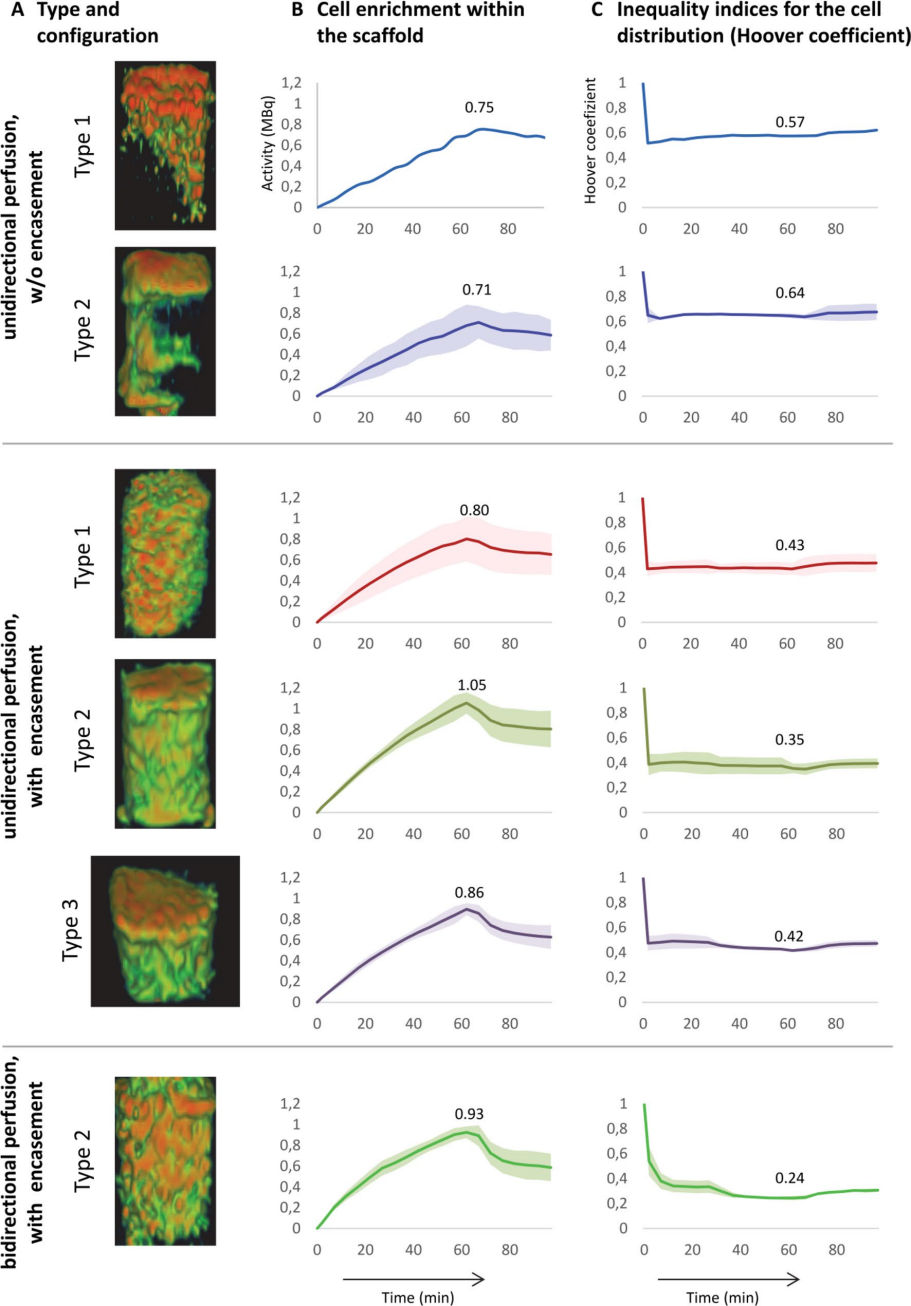
Cell enrichment and distribution within the bioreactor during perfusion. (**A**) Micro-PET/CT images of [^18^F]FDG-labeled stem cells after 60 min perfusion. Cells are marked in green and red representing only a few cells and many cells, respectively, depending on cell enrichment. The scaffolds are masked. Obviously, cells attach primarily in the upper half of the scaffold. In bioreactor type 2 w/o encasement (type2_enc−), cells tend to bypass the edge of the scaffold (2nd row). Note, that bioreactor types were sealed with silicone in later experiments (rows 3 to 6). Combining type 2 with encasement and unidirectional perfusion resulted in the highest cell enrichment within the scaffold block (4th row), but changing unidirectional to an oscillating perfusion mode resulted in the most homogenous cell distribution (6th row). (**B**, **C**) Graphical representation of cell accumulation and distribution in the entire scaffold during 60 min perfusion with cell suspension followed by 40 min wash out phase. (**B**) Cell enrichment was measured by the activity of [^18^F]FDG-labeled stem cells within the scaffold. (**C**) Homogeneity of cell distribution is represented by the Hoover coefficient. Values close to zero indicate a uniform distribution. Notably, the bioreactor type 2 with encasement (type2_enc+) harbors the highest cell enrichment and type 2 with encasement and oscillating perfusion (type2_enc+_osc) the lowest Hoover coefficient. Numbers in the graph specifies peak value of activity as well as Hoover coefficient at 60 min.

The micro-PET/CT examinations revealed distinct differences in the efficiency of cell seeding for the different bioreactor configurations and perfusion algorithms in terms of enrichment and distribution of cells. The residual activity (in MBq) was taken as a measure for the seeding efficiency within the scaffold block. Regarding all bioreactor configurations, a linear, but differently steep increase in activity over the scaffold block during the perfusion with cell suspension for 60 min was determined, followed by a slight decrease with reaching a plateau during the 40 min wash-out phase due to outwash of non-adherent cells and [^18^F]FDG efflux from the chamber and the seeded cells (Fig. 7B). In case of bioreactors without press-fit silicone encasement, bioreactor type1_enc− (1 mm cylindrical inflow and outflow) and bioreactor type2_enc− (15 × 15 mm conical shaped inflow and outflow) showed comparable cell enrichment in terms of cell activity of 0.75 MBq (in a single experiment) and 0.71 MBq (n = 2), respectively. In general, a shift of the cell stream towards the edge of the scaffold could be shown in both bioreactor types (Fig. 7A), so it can be assumed that cells were not perfused through the scaffold block but rather flowed past the edge. A sealing of the lateral surface of the scaffolds by silicone encasement resulted in a more optimized flow through the scaffold. The bioreactor type1_enc+ achieved a significantly higher cell enrichment (0.80 ± 0.18 MBq) after approx. 60 min of perfusion, and at the same time a lower Hoover coefficient indicating a higher homogeneity of cell distribution within the scaffold block (Fig. 7B, Table 2). The best results were recorded with the conical diffuser and encasement (1.05 ± 0.10 MBq). However, the PET/CT images show that unidirectional perfusion could only reliably seed the upper regions of the scaffold homogeneously (Fig. 7A). A further modification (bioreactor type3_enc+), in which the orientation of the scaffold block was changed, and the flow went from one lateral surface to the opposite side (using a 15 × 25 mm diffuser at the inflow and the outflow and a silicone encasement) yielded in a worse efficiency and homogeneity of cell seeding (Fig. 7, 5th row). Therefore, an oscillating bidirectional perfusion was additionally tested (bioreactor type2_enc+ _osc). By changing the direction of perfusion after 30 min, a homogeneous cell distribution over the entire scaffold block was achieved (Fig. 7A, bottom row). The Hoover coefficient was lowered to 0.24 indicating an almost uniform distribution, which represents a significant improvement compared to all other tested configurations (Fig. 7B, Table 2). Only by using the bioreactor type2_enc+ _osc combining silicone encasement and oscillating perfusion mode, a homogeneous stem cell distribution could be accomplished after 60 min perfusion within the entire scaffold (Fig. 7A).

**Table 2:**
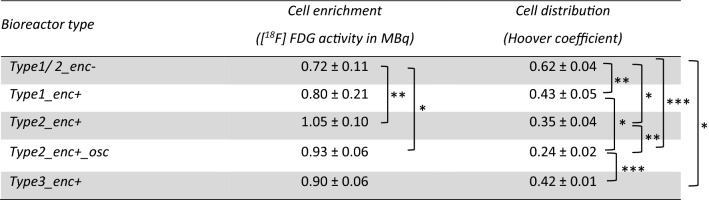
Measurement of cell enrichment and homogeneity of the cell distribution within different bioreactor types by dynamic micro-PET/CT after 60 min perfusion.

Cell enrichment was calculated by detecting [^18^F]FDG-labeled cells. Homogeneity of cell distribution was presented by calculating the Hoover coefficient, whereby a lower Hoover coefficient indicates higher homogeneity of cell distribution in the scaffold (mean ± SD, student’s t test, *P < 0.05, **P < 0.01, ***P < 0.001 considered significantly different compared to the respective bioreactor type).

To validate PET/CT images of cell seeding in terms of cell distribution, scanning electron microscopic (SEM) analysis of scaffold blocks were performed using the bioreactor type2 with encasement and unidirectional perfusion (type2_enc+) exemplarily. In general, images taken in the direction of perfusion flow confirmed a successful cell seeding (Supplementary Fig. 4). Immediately after perfusion (t_0_), fine pores of the scaffold block were covered with initially attached cells at all three positions (inflow, middle and outflow region of the block) (Supplementary Fig. 4A). An increasing adherence and spreading of cells on the scaffold surface could be observed already 3 h after perfusion. However, by unidirectional perfusion only sparsely distributed cells were found 3 h after perfusion when scanning was performed in opposite direction of the perfusion flow, nearly independent of the position within in the scaffold block (Supplementary Fig. 4C). This means that the cells are well adhered to the scaffold surfaces in the direction of perfusion, but have not yet migrated to the scaffold that faces away from the perfusion. Obviously, a sufficient cell seeding could not be achieved in the outflow region as well as in some areas within the scaffold block by using an unidirectional perfusion bioreactor setting. Only by applying the oscillating perfusion mode a homogeneous (lowest Hoover coefficient: 0.24 ± 0.02) stem cell distribution was guaranteed (Fig. 7, Table 2).

### Osteogenic differentiation of ASC in long-term perfusion experiments

**Figure 8:**
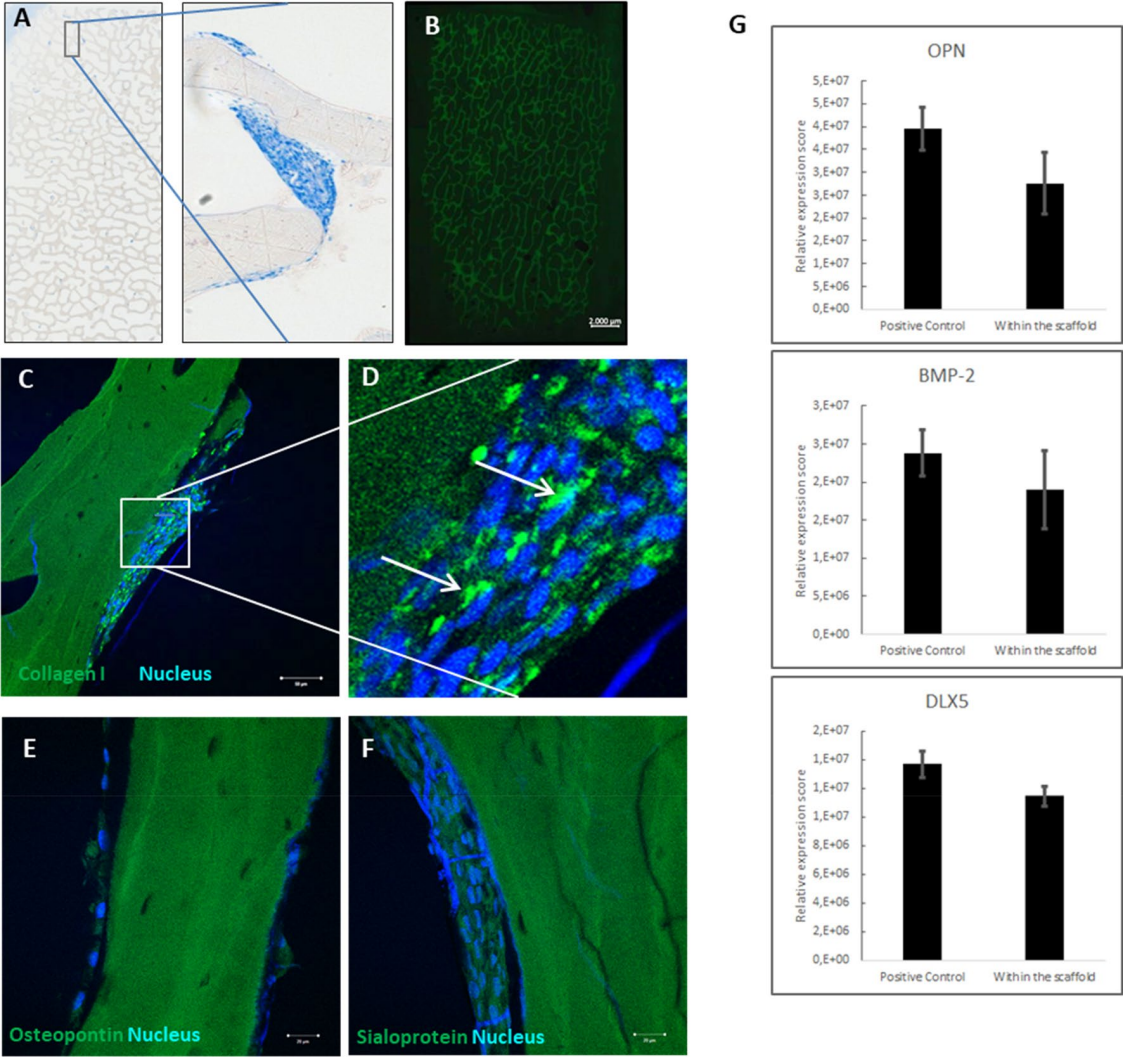
Histological analysis of scaffolds perfused with oscillating perfusion mode. Scaffold blocks were perfused in a bioreactor type2_enc+_osc with 0.5 × 10^6^ cells/ml, followed by 14-day incubation in a roller culture. (**A**) Scaffolds were stained with Giemsa's Azure-Eosin-Methylene Blue Solution and Toluidine Blue-Methylene Blue-Pyronine solution showing multi-layered stem cell accumulations extending over the surface and the pores of the bone graft substitute. (**B**) Autofluorescence image of the scaffold block representing background fluorescence visualized by fluorescence microscopy equipped with FITC filter set. Immunohistochemical staining of osteogenic differentiation markers (green) Collagen I (**C, D**), Osteopontin (**E**) and Bone Sialoprotein (**F**) counterstained with Hoechst (blue) to visualize cell nuclei by confocal LSM. White arrows represent newly expressed marker proteins by ASC. Collagen I is expressed in all stem cell layers as well as Osteopontin and Bone Sialoprotein, indicating differentiation of ASC into the osteogenic lineage (LSM 780, Carl Zeiss Microscopy GmbH). (**G**) Expression analysis of three osteogenic marker genes (i) osteopontin (OPN), also known as bone sialoprotein I, (ii) bone morphogenetic protein 2 (BMP-2), and (iii) the distal-less homeobox 5 gene (DLX-5). Notably, transcript levels of all three osteogenic differentiation genes do not differ significantly between the positive control (chemically differentiated ASCs) and the perfused cells within the scaffold.

Because a homogeneous cell distribution was achieved by an oscillating bidirectional perfusion, bioreactor type 2 with encasement and oscillating perfusion (type2_enc+_osc) was used for testing the osteogenic differentiation of ASC in long-term perfusion experiments lasting 14 days. Analysis of the differentiation properties of ASC within the scaffolds proved a forced osteogenic differentiation shown by osteogenic marker expression on protein level by immunohistochemical analysis (Fig. 8C–F) and on transcript level by RT-PCR (Fig. 8G). In general, ASC were grown in multi-layers which were extended over the surface and pores of the bone graft substitute (Fig. 8A). Autofluorescence of the scaffold is shown in Fig. 8B. A high collagen I expression and enrichment within the cell layer was marked by green fluorescence labeling (Fig. 8C). 3D reconstructed cell layers confirmed that the cells were connected and kind of embedded by the collagen I protein. Furthermore, the expression of specific osteogenic differentiation marker like osteopontin and bone sialoprotein indicates that the perfused ASCs are able to differentiate into the osteogenic direction (Fig. 8D, E). Compared to chemically differentiated ASC (positive control) the stem cells within the scaffold, 14 days after perfusion showed a marginally lowered, but not significant altered gene expression of three selected osteogenic differentiation markers: osteopontin (OPN), bone morphogenetic protein 2 (BMP2), distal-less homeobox 5 gene (DLX5).

## Discussion

Although the treatment of bone defects in combination with stem cell transplantation and scaffolds works in principle, there are hardly any clinically practicable procedures^24–26^. This is partly due to fundamental regulatory problems, but also to the lack of reproducibility of seeding strategies, especially if you consider the scale-dependent pore distribution within the scaffold and the related structural heterogeneity features^27^. For regulatory reasons, a chairside procedure is mandatory, which in turn means that the seeding process should be limited to a working time frame of 30–60 min. In addition, a closed system is important, and a quite homogeneous seeding of the scaffold must be ensured even with complex scaffold geometries. By automating and standardizing tissue manufacture in controlled closed systems, bioreactors could reduce production costs and allow effective colonization strategies^18^. In our experiments, we could show that mathematical modeling is able to predict the homogeneity and efficiency of seeding depending on defined parameters such as bioreactor geometry, perfusion rate and cell density of the perfusate. A particular challenge poses the imaging of seeded cells in opaque scaffolds, which could be reliably realized with dynamic micro-PET/CT. Compared to conventional histological or laser scanning microscopic imaging, this method saves time and allows a higher throughput of experiments. To our knowledge, micro-PET/CT was used in this context for the first time and has been proven to be an efficient way to test various seeding strategies by using a standard cuboidal scaffold. Even though Porter et al. also used micro-PET / CT for monitoring, our approach differs in that we did not track the mineralization within the scaffold but the colonization efficiency and homogeneity of the stem cells in the bone substitute material^28^. However, the option behind the in silico modeling would be the extrapolation to various anatomically or individually shaped scaffold geometries.

By testing different geometries for the in- and outlets of the bioreactor, the conical form proved to be the most effective for homogeneous colonization. Furthermore, the encasement of scaffolds plays an important role to prevent leakage flow around the scaffolds. Consequently, a silicone encasement for individually structured scaffolds can be generated, as it was already implemented by the working group of Huber et al.^16^. However, combining the conical inlets with custom made silicone encasement resulted in significant homogeneously distributed cell seeding, at least for the upper half of the scaffold using unidirectional perfusion. If an oscillating perfusion is additionally used, the homogeneity of the cell distribution can be further increased resulting in a low Hoover coefficient of 0.24, an improvement already mentioned by Wendt et al.^29^. A further advantage was the use of 3D printing for the manufacture of the bioreactor, as 3D printing today is the most efficient way to create new geometries quickly and flexibly in personalized healthcare. Several direct perfusion bioreactors have been designed to engineer bone substitutes by using various cell types and materials^30–33^. By the way, these and many other studies demonstrate the advantage of a perfusion culture compared to a static culture to colonize bone replacement materials with human cells over a period of time up to 35 days. Long-term cultivation was important for the present research question insofar as a differentiation of the seeded ASC into the osteogenic lineage could be demonstrated. Of course, ASCs isolated and cultivated in the laboratory only served as a model for cells that might be obtained simultaneously in the same procedure, i.e., bone marrow derived stem cells, or, depending on future regulatory developments, simultaneously harvested adipogenic stem cells. Therefore, it has to be conceded that these results cannot be transferred unconditionally to the in clinical situation. Especially when you consider the limitations of our experiments. After all, both the scaffold geometry and the variability of the pore size and distribution are decisive parameters for homogeneous colonization with cells. Nevertheless, with our experiments we have laid the foundation for effective monitoring of cell colonization within a scaffold. We are currently working on the colonization of clinic-related scaffold geometries and are evaluating the healing of colonized blocks in a rat model.

In summary, this study shows that an individual bioreactor can be easily and quickly generated with a conventional 3D printing process, which can also be adapted to the individual geometry of the patient's bone substitute material. This bioreactor is suitable for homogeneous seeding of individual scaffolds with stem cells in less than an hour. The following four parameters were decisive for efficient cell colonization of the scaffold in the present investigations: 1. cell concentrations of 0.5 to 1.0 × 10^6^ cells/ml, 2. a low perfusion rate of 0.5–1 ml/min, 3. scaffold encasement with a silicone sleeve, and 4. an oscillating perfusion mode, which was already recommended by Du et al.^34^. In addition, the central concern of the study was to test the usability of micro-PET/CT for the validation of seeding algorithms that were previously modeled in silico. In fact, the three-dimensional distribution of the cells in opaque ceramic scaffolds can only be determined by means of complex histological procedures. Optical methods are not applicable or only on a small scale with low penetration depth. Even imaging, e.g. micro-CT or micro-MRI cannot provide such data. The experiments have shown that the non-destructive micro-PET/CT examination is a reliable, reproducible and efficient method to determine the three-dimensional distribution of seeded cells within the scaffolds. The results were congruent with those obtained by histological processing combined with automated reconstruction. Thus, micro-PET/CT examination is a method that can be used rather easily and efficiently to prove the validity of a seeding procedure and to objectively compare seeding algorithms. In addition, it is non-destructive and, in contrast to all other available methods, provides a unique impression of the seeding process over time. In principle, it is therefore also suitable for quality control. To our knowledge, this study also shows the first time that micro-PET-based analysis is equivalent to fluorescence-based, histological evaluation with regard to the cellular distribution within a scaffold.

## Conclusion

In view of potential future chairside applications, the seeding process of scaffolds (ceramic bone blocks) must be fast and efficient and should be able to provide homogeneous cell distributions. The modeling of fluid mechanics in silico is suitable for the preliminary testing of perfusion algorithms and bioreactor designs to achieve this goal for specific scaffold geometries. Dynamic [^18^F]FDG micro-PET/CT visualization allows monitoring of the seeding process in opaque ceramic scaffolds in a non-destructive manner and the quantification of seeding efficiency and homogeneity. Conical connector design, lateral sealing of the scaffold e.g., by a silicone encasement, a low stem cell concentration of approx. 0.5 × 10^6^ cells/ml in the perfusate, a low perfusion rate of 0.5 ml/min and an oscillating perfusion mode improves efficiency and homogeneity of cell seeding. By optimizing the bioreactor design and fabrication using 3D printing, the efficiency and homogeneity of the seeding process is significantly improved, and, under these conditions, the homogenous seeding of scaffolds may be performed within a time frame of 60 min.

## Supplementary Information

Supplementary Information.

## Data Availability

The datasets generated during and/or analyzed used in this manuscript are available from the first and corresponding author on reasonable request.

## References

[1] Hara ES, Okada M, Nagaoka N, Nakano T, Matsumoto T (2021). Re-Evaluation of initial bone mineralization from an engineering perspective. Tissue Eng. Part B Rev..

[2] Fishero BA, Kohli N, Das A, Christophel JJ, Cui Q (2015). Current concepts of bone tissue engineering for craniofacial bone defect repair. Craniomaxillofac. Trauma Reconstr..

[3] Bourlier V (2008). Remodeling phenotype of human subcutaneous adipose tissue macrophages. Circulation.

[4] Ansari M (2019). Bone tissue regeneration: Biology, strategies and interface studies. Prog. Biomater..

[5] Ho-Shui-Ling A (2018). Bone regeneration strategies: Engineered scaffolds, bioactive molecules and stem cells current stage and future perspectives. Biomaterials.

[6] Yamada S, Yassin MA, Schwarz T, Hansmann J, Mustafa K (2021). Induction of osteogenic differentiation of bone marrow stromal cells on 3D polyester-based scaffolds solely by subphysiological fluidic stimulation in a laminar flow bioreactor. J. Tissue Eng..

[7] Solakoglu Ö (2019). Improved guided bone regeneration by combined application of unmodified, fresh autologous adipose derived regenerative cells and plasma rich in growth factors: A first-in-human case report and literature review. WJSC.

[8] Sauerbier S (2010). In vivo comparison of hard tissue regeneration with human mesenchymal stem cells processed with either the FICOLL method or the BMAC method. Tissue Eng. Part C Methods.

[9] Smiler D, Soltan M, Lee JW (2007). A histomorphogenic analysis of bone grafts augmented with adult stem cells. Implant Dent..

[10] Soltan M, Smiler DG, Gailani F (2005). A new "platinum" standard for bone grafting: Autogenous stem cells. Implant Dent..

[11] Moreno Sancho F (2019). Cell-based therapies for alveolar bone and periodontal regeneration concise review. Stem Cells Transl. Med..

[12] Patel N, Kim B, Zaid W, Spagnoli D (2017). Tissue engineering for vertical ridge reconstruction. Oral Maxillofac. Surg. Clin. North Am..

[13] Alonso-Goulart V (2021). Biomaterials and adipose-derived mesenchymal stem cells for regenerative medicine: A systematic review. Materials (Basel, Switzerland).

[14] Wongchuensoontorn C (2009). Application of a new chair-side method for the harvest of mesenchymal stem cells in a patient with nonunion of a fracture of the atrophic mandible–a case report. J. Cranio-Maxillo-Fac. Surg. Off. Publ. Eur. Assoc. Cranio-Maxillo-Fac. Surg..

[15] Weinzierl K, Hemprich A, Frerich B (2006). Bone engineering with adipose tissue derived stromal cells. J. Cranio-Maxillo-Fac. Surg. Off. Publ. Eur. Assoc. Cranio-Maxillo-Fac. Surg..

[16] Schmid J (2018). A perfusion bioreactor system for cell seeding and oxygen-controlled cultivation of three-dimensional cell cultures. Tissue Eng. Part C Methods.

[17] Sladkova M, de Peppo G (2014). Bioreactor systems for human bone tissue engineering. Processes.

[18] Martin I, Wendt D, Heberer M (2004). The role of bioreactors in tissue engineering. Trends Biotechnol..

[19] Carpentier B, Layrolle P, Legallais C (2011). Bioreactors for bone tissue engineering. Int. J. Artif. Organs.

[20] Ott R (2017). Experimental and numerical investigations of fluid flow for optimized in vitro stem cell loading in xenografts. Curr. Direct. Biomed. Eng..

[21] Frerich B, Winter K, Scheller K, Braumann U-D (2012). Comparison of different fabrication techniques for human adipose tissue engineering in severe combined immunodeficient mice. Artif. Organs.

[22] Zimmerlin L (2010). Stromal vascular progenitors in adult human adipose tissue. Cytometry. Part A J. Int. Soc. Anal. Cytol..

[23] Kasten A (2014). Comparative in vitro study on magnetic iron oxide nanoparticles for MRI tracking of adipose tissue-derived progenitor cells. PLoS ONE.

[24] Yeatts AB, Fisher JP (2011). Bone tissue engineering bioreactors: dynamic culture and the influence of shear stress. Bone.

[25] Torres-Guzman, R. A. *et al.* Application of human adipose-derived stem cells for bone regeneration of the skull in humans. *J. Craniofac. Surg.* **Publish Ahead of Print** (2021). 10.1097/SCS.0000000000008114 34636755

[26] Kirankumar, S. *et al.* Modern approaches on stem cells and scaffolding technology for osteogenic differentiation and regeneration. *Exp. Biol. Med. (Maywood),* 15353702211052927 (2021). 10.1177/15353702211052927 PMC8919323 34648374

[27] Massai D (2014). Image-based three-dimensional analysis to characterize the texture of porous scaffolds. BioMed Res. Int..

[28] Porter BD, Lin ASP, Peister A, Hutmacher D, Guldberg RE (2007). Noninvasive image analysis of 3D construct mineralization in a perfusion bioreactor. Biomaterials.

[29] Wendt D, Marsano A, Jakob M, Heberer M, Martin I (2003). Oscillating perfusion of cell suspensions through three-dimensional scaffolds enhances cell seeding efficiency and uniformity. Biotechnol. Bioeng..

[30] Klein-Nulend J, Bakker AD, Bacabac RG, Vatsa A, Weinbaum S (2013). Mechanosensation and transduction in osteocytes. Bone.

[31] Grayson WL (2010). Engineering anatomically shaped human bone grafts. Proc. Natl. Acad. Sci. U.S.A..

[32] Chen M (2011). A modular approach to the engineering of a centimeter-sized bone tissue construct with human amniotic mesenchymal stem cells-laden microcarriers. Biomaterials.

[33] Bjerre L, Bünger CE, Kassem M, Mygind T (2008). Flow perfusion culture of human mesenchymal stem cells on silicate-substituted tricalcium phosphate scaffolds. Biomaterials.

[34] Du D, Furukawa KS, Ushida T (2009). 3D culture of osteoblast-like cells by unidirectional or oscillatory flow for bone tissue engineering. Biotechnol. Bioeng..

