# Supplementary material for: An optimized 3D-printed perfusion bioreactor for homogeneous cell seeding in bone substitute scaffolds for future chairside applications
Source: Sci Rep. 2021 Nov 15;11:22228. doi: 10.1038/s41598-021-01516-8 (PMC8593024; doi:10.1038/s41598-021-01516-8)
Supplement: Supplementary file 1 — Supplementary Information. [file 41598_2021_1516_MOESM1_ESM.pdf]

Supplemental figure 1

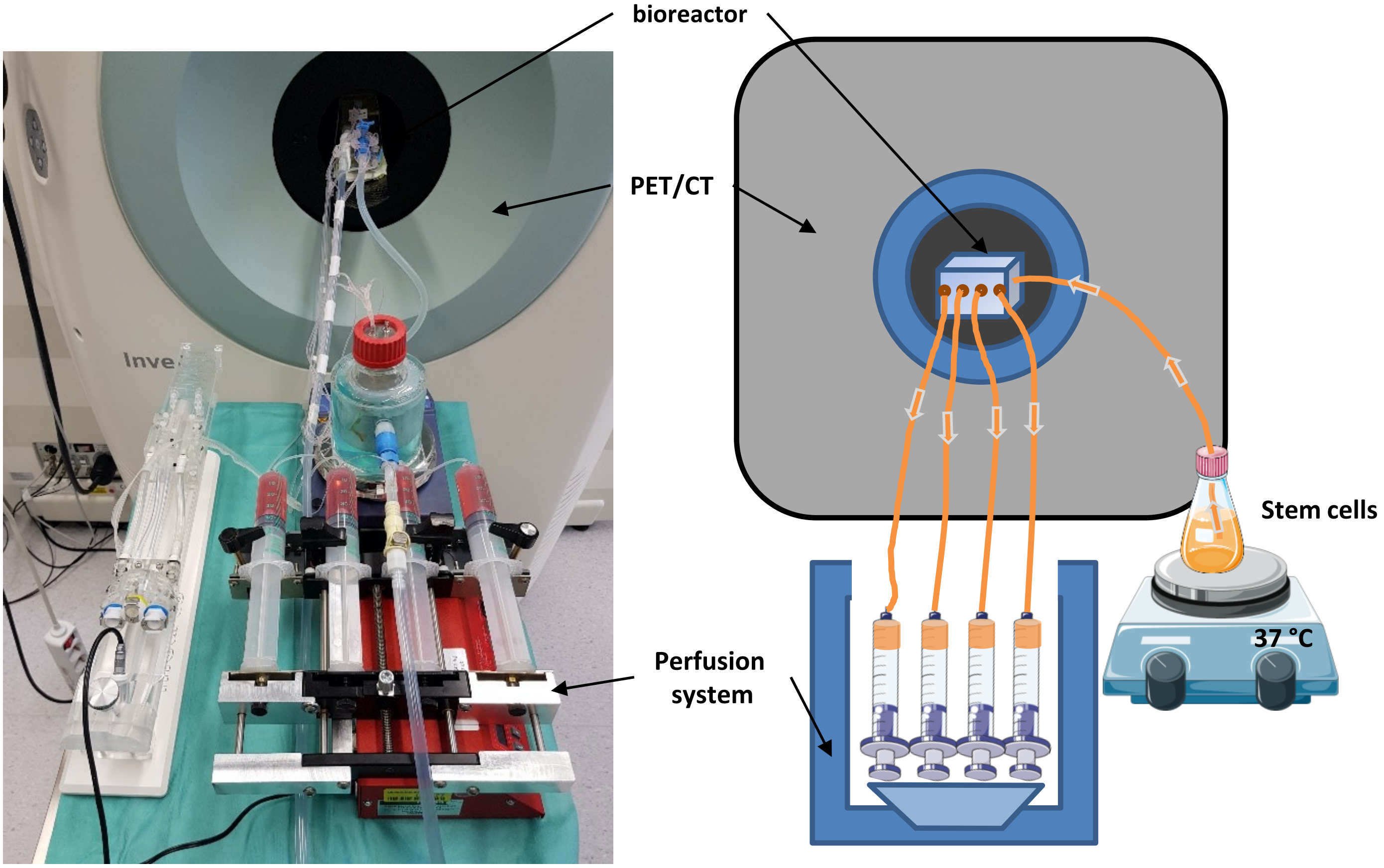

Supplemental Fig. 1 Scheme of the perfusion system used in the micro-PET-CT experiments. Created with Biorender.

Supplemental figure 2

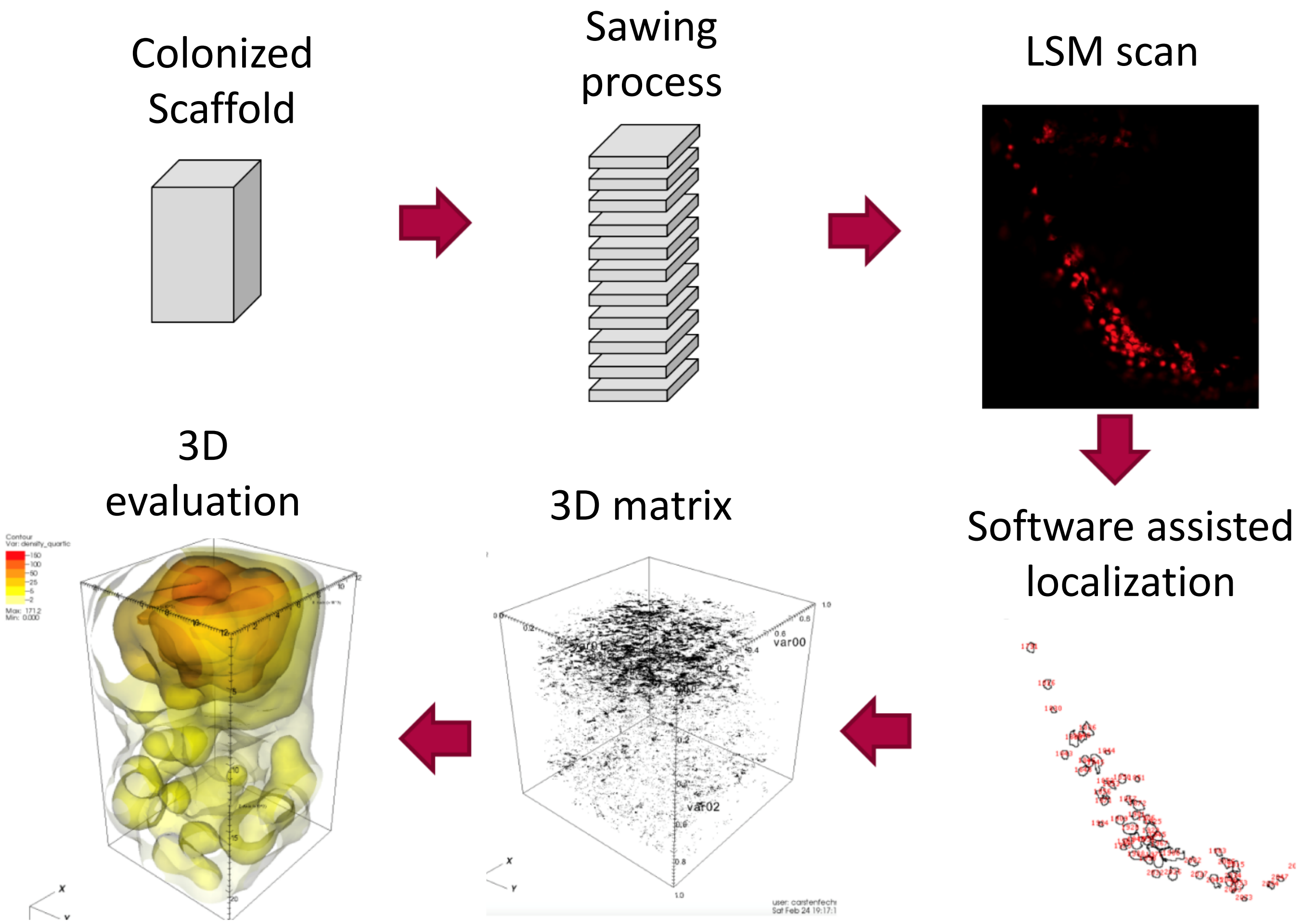

Supplemental Fig. 2 Schematic representation of the process of three-dimensional representation of cell distribution in the xenograft block using LSM on 1.5 mm thick slices, image acquisition in LSM and assignment of coordinates with FIJI, subsequent three-dimensional reconstruction with VisLT.

Supplemental figure 3

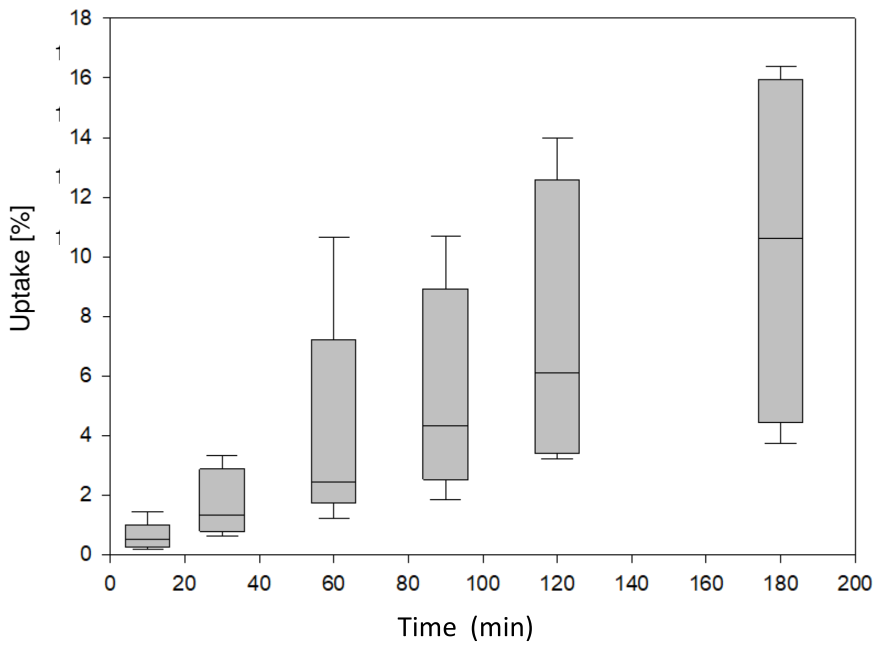

Supplemental Fig. 3 Uptake of [18F]FDG in ASC related to incubation time (n=6).

Supplemental figure 4

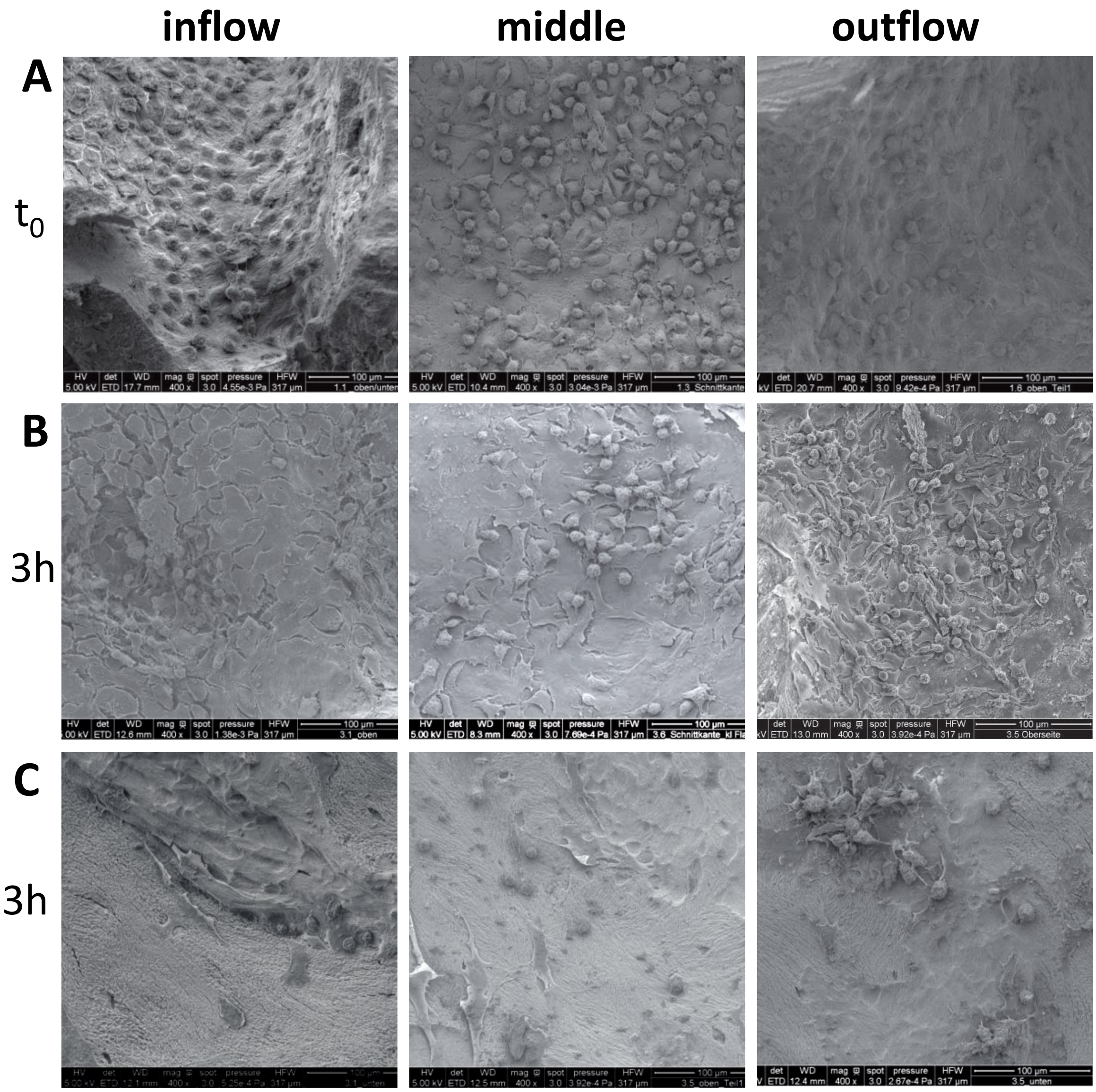

Supplemental Fig. 4 Scanning electron micrographs of cell adherence after unidirectional perfusion in bioreactor type2 with encasement (type2_enc+). Images were taken in direction of perfusion flow (A) immediately after perfusion t_0 and (B) after incubation of 3h as well as (C) averted to flow direction. At all-time points, images from the inflow, middle, and outflow regions are shown. Whereas immediately after perfusion cells were only initially attached, cells spread and formed a cell monolayer already 3h after perfusion. In contrast, only a few cells are attached averted to flow direction 3h after perfusion
